# Anomalous Origin of the Left Coronary Artery From the Right Pulmonary Artery (ALCARPA): A Case Report

**DOI:** 10.7759/cureus.87762

**Published:** 2025-07-12

**Authors:** Sachin Talwar, Srikant Sharma, Bharat Siddharth, Vishal V Bhende

**Affiliations:** 1 Cardiothoracic and Vascular Surgery, All India Institute of Medical Sciences, New Delhi, IND; 2 Cardiothoracic and Vascular Surgery, The Mission Hospital, Durgapur, IND; 3 Cardiovascular Surgery, Aster Ramesh Hospitals, Guntur, IND; 4 Pediatric Cardiac Surgery, Bhanubhai and Madhuben Patel Cardiac Centre, Shree Krishna Hospital, Bhaikaka University, Anand, IND

**Keywords:** anomalous left coronary artery from the right pulmonary artery (alcarpa), congenital heart disease, coronary vessel anomalies, intramural coronary artery course, myocardial ischemia

## Abstract

Although the congenitally anomalous left coronary artery (LCA) is a rare condition, it can give rise to a most rare case, which is an anomalous LCA from the right pulmonary artery (RPA) (ALCARPA). If left untreated, ALCARPA results in myocardial territories receiving low-pressure, oxygen-poor blood. A male infant aged eight months exhibited signs of respiratory distress, poor feeding, and precordial activity. The diagnosis of ALCARPA is usually considered in infants with otherwise unexplained left ventricular dysfunction or mitral valve regurgitation. Imaging studies, including computed tomography angiography and echocardiography, showed a markedly dilated right coronary artery and a dilated left ventricle, confirming ALCARPA. Using autologous pericardium treated with glutaraldehyde, the abnormal LCA was directly anastomosed to the aorta. Postoperative recovery was uneventful, with elective inotropic support provided. To prevent fatal arrhythmias and myocardial ischemia, prompt diagnosis and surgical creation of a dual-coronary system are essential.

## Introduction

Coronary arteries (CA) commonly emerge from the aortic sinuses and deliver oxygen-rich blood to the heart muscle. About one out of every 300,000 live newborns has an aberrant left coronary artery (LCA) that originates from the main pulmonary artery (MPA) (ALCAPA) [1]. Additionally, the right pulmonary artery (RPA) may give rise to an anomalous LCA (ALCARPA). Consequently, myocardial territories perfused by this anomalous vessel (ALCARPA) are susceptible to inadequate blood flow and ischemia, particularly during conditions that elevate cardiac metabolic demands. The clinical severity of ALCARPA is determined by the magnitude of the left-to-right shunt through collateral arteries and the extent of collateral blood flow from the right coronary artery (RCA) [1-3]. Among pediatric patients, ALCAPA is the leading cause of myocardial ischemia and infarction. Without surgical correction, mortality during infancy can reach up to 90% within the first year of life [2]. ALCARPA is even rarer, with fewer than 50 reported cases worldwide [1]. Here, we report a case of ALCARPA, which was successfully reimplanted into the aorta.

## Case presentation

A male infant of eight months had a history of poor feeding and was in respiratory distress with increased precordial activity. Clinical examination revealed normal heart sounds without a murmur, bilateral crepitations on lung auscultation, and hepatomegaly. The patient was initially managed conservatively with diuretics and underwent further evaluation.

Chest radiography demonstrated cardiomegaly and signs of severe pulmonary venous hypertension. Electrocardiography revealed prominent Q waves in lead I, aVL, and V5-V6. A dilated and malfunctioning left ventricle (LV) with an ejection fraction of 10%-15% as revealed on two-dimensional echocardiography (ECHO). Furthermore, the mitral valve's anterolateral papillary muscle was echogenic, exhibiting mild mitral regurgitation, a dilated RCA, and ALCARPA.

**Figure 1:**
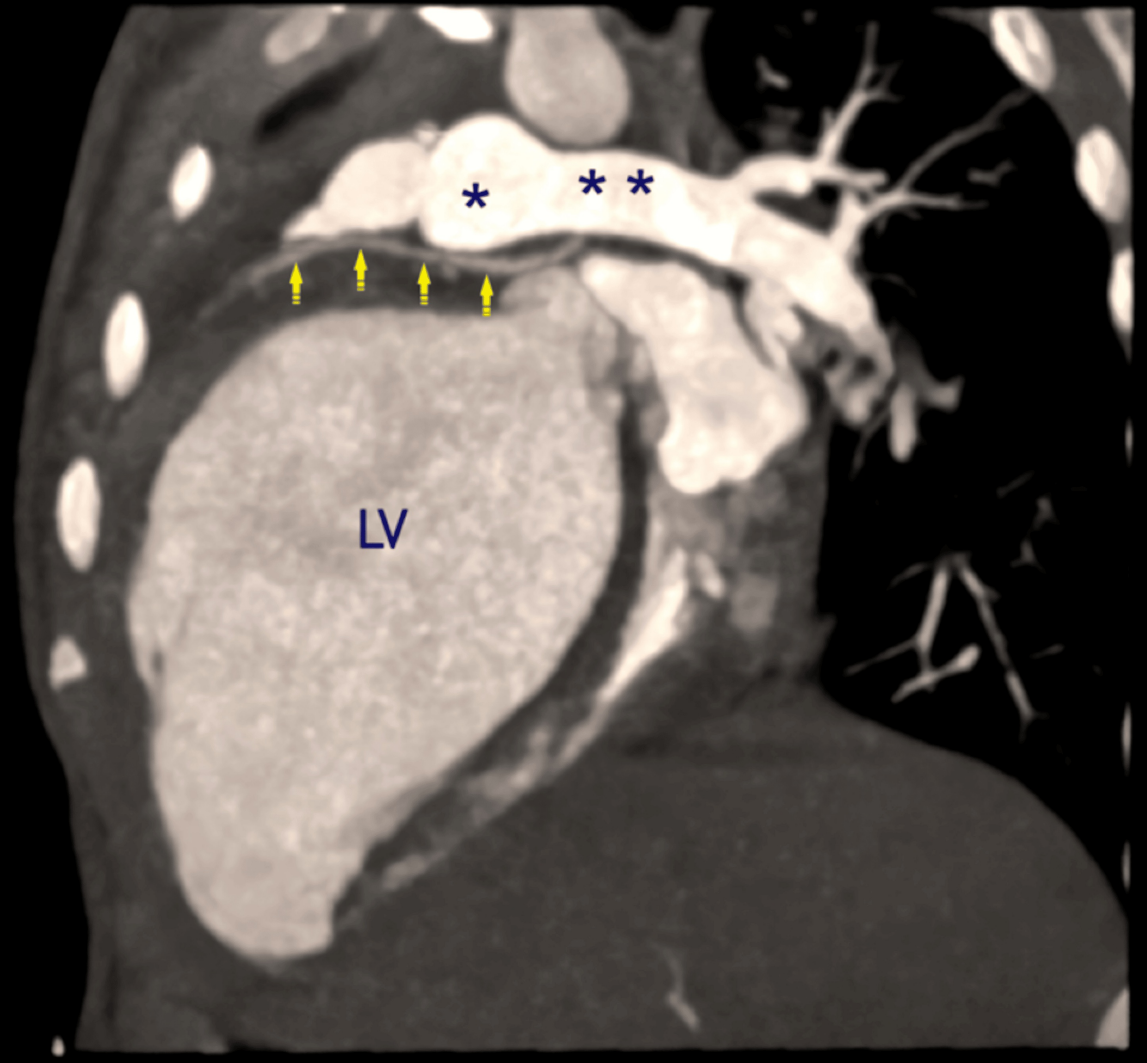
The oblique sagittal maximum intensity projection (MIP) reconstruction of CT angiography reveals that the LCA started abnormally from the undersurface of the RPA (ALCARPA, yellow arrows) with a dilated left ventricle (LV). **, RPA, right pulmonary artery; *, MPA, main pulmonary artery; CT, computed tomography Image Credits: Dr. Sachin Talwar

**Figure 2:**
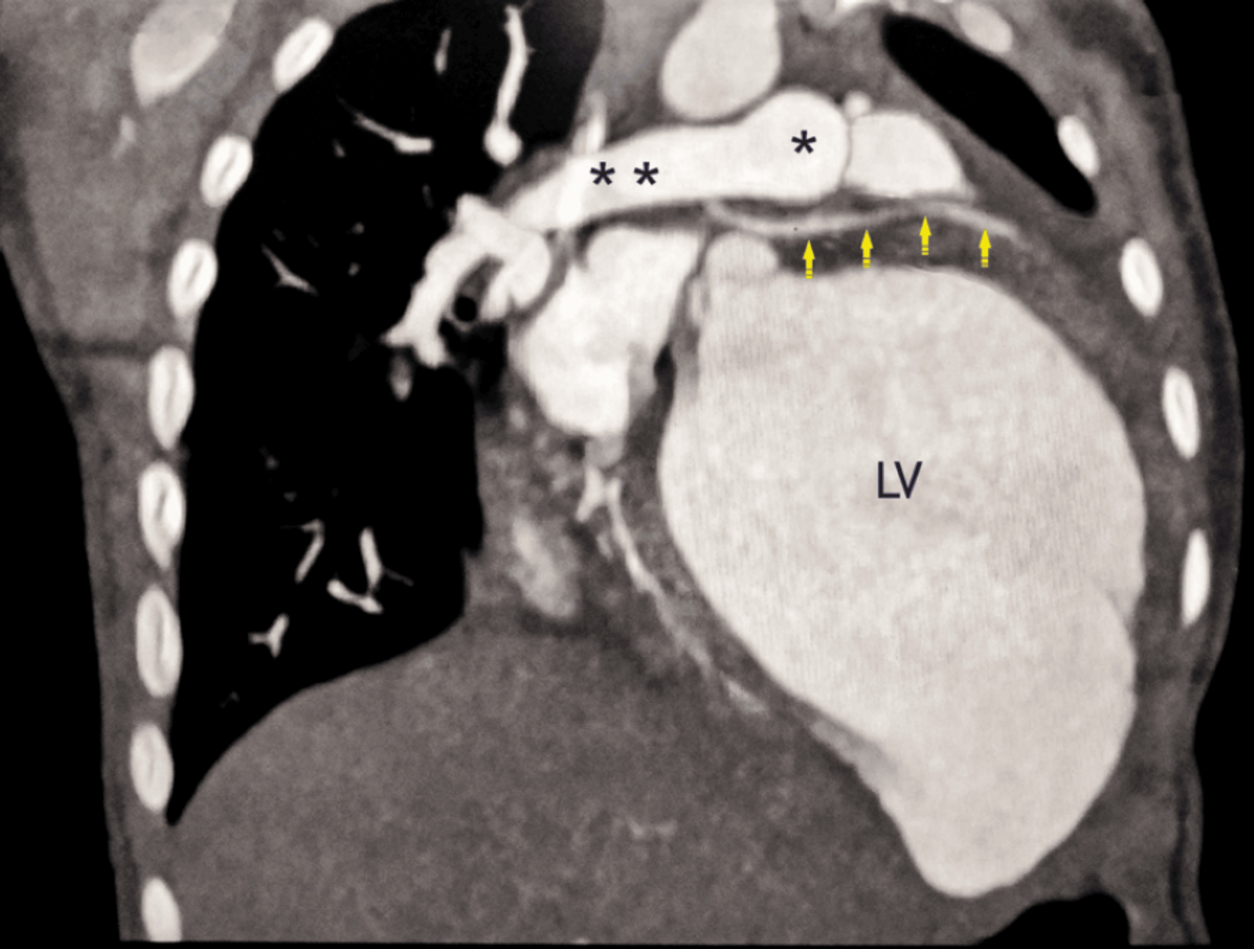
CT angiography with oblique coronal maximum intensity projection (MIP) reconstruction showing the abnormal origin of the LCA from the underside of the RPA (ALCARPA, yellow arrows) with a dilated left ventricle (LV). **, RPA, right pulmonary artery; *, MPA, main pulmonary artery; CT, computed tomography Image Credits: Dr. Sachin Talwar

The LCA originating from the RPA, which is situated 5 mm distal to the pulmonary trunk's bifurcation, was validated by computed tomographic (CT) angiography. The examination further revealed that the left anterior descending artery (LAD), which arose from the ALCARPA, was normal, with the accessory LAD following a prepulmonic course. The RCA was visibly dilated (Figures 1-2).

The LCA was directly reimplanted into the aorta via a conventional median sternotomy. For later usage, autologous pericardial tissue was harvested and treated with 0.625% glutaraldehyde. Snares were positioned around the dissected left pulmonary artery (LPA) and RPA. After aorto-bicaval cannulation, a standard moderately hypothermic (28°C) (CPB) was started, with simultaneous tightening of the snares around both LPA and RPA in order to prevent LCA blood flow from being diverted to the lungs.

After aortic cross-clamping, left ventricular decompression was achieved by inserting a vent into the right pulmonary vein. Del Nido cardioplegia (30 mL/kg) was then given through the aortic root. Upon transecting the aorta, the left coronary ostium was absent within its lumen. The ostium of the anomalous LCA, which is situated on the posterior-inferior part of the RPA immediately distal to its division, was exposed when the pulmonary trunk was cut proximal to its bifurcation. A probe passed into the ostium showed that the initial 1 cm of the LCA was intramural within the RPA wall, which was not evident on preoperative CT.

Careful dissection was performed to excise the main LCA from the RPA, preserving the integrity of its intramural course. After sufficient mobilization of the excised LCA with its surrounding button of tissue, at the medial base of the nearby left coronary sinus of the aorta, a trapdoor incision was made. The mobilized coronary button was then anastomosed to this site using a 7/0 polypropylene suture. The aorta was reconstructed by anastomosing the proximal and distal segments.

The defect in the RPA created by the LCA excision was repaired using a patch of the glutaraldehyde-treated pericardium, and continuity between the transected MPA segments was restored. After 190 minutes, CPB weaning was feasible with optional inotropic support that included an equal amount of adrenaline and milrinone (0.05 mcg/kg/min) and dopamine (5 mcg/kg/min). The aortic cross-clamp time was 56 minutes. The left atrial pressure, monitored via a left atrial line, was 12 mmHg upon termination of CPB.

The child was extubated on day five post surgery to a high-flow nasal cannula, which was gradually weaned over the next week, and the child was discharged after 20 days in stable condition with near‐normal ejection fraction and mild mitral regurgitation, maintaining room air saturations of near 100% with the advice of regular follow-ups. The child is doing well at the four-month follow-up clinic visit and was thriving normally with pulmonary artery pressures half-systemic and good bi-ventricular function.

Consent for publication of these case details was obtained from the patient’s legally authorized representative, who provided consent to publish this anonymized case report following ethical committee approval.

## Discussion

ALCAPA was first described in 1866 by Brooks [4]. The first clinical description in conjunction with autopsy findings was described by Bland et al. in 1933 [2]. Later, Edwards subsequently described the pathophysiology of blood flow in CA [3], so the anomaly is also called Bland-White-Garland syndrome. The ALCAPA anomaly may result from (a) abnormal septation of the conotruncus into the aorta and pulmonary artery or from (b) persistence of the pulmonary buds together with involution of the aortic buds that eventually form the coronary arteries [5].

During the neonatal period, as MPA pressure drops, there is an increase in left-to-right shunting across the coronary circulation, which can cause a coronary steal and myocardial ischemia [3,6,7]. Intercoronary collaterals from an enlarging RCA ultimately supply the myocardial territories [3].

**Figure 3:**
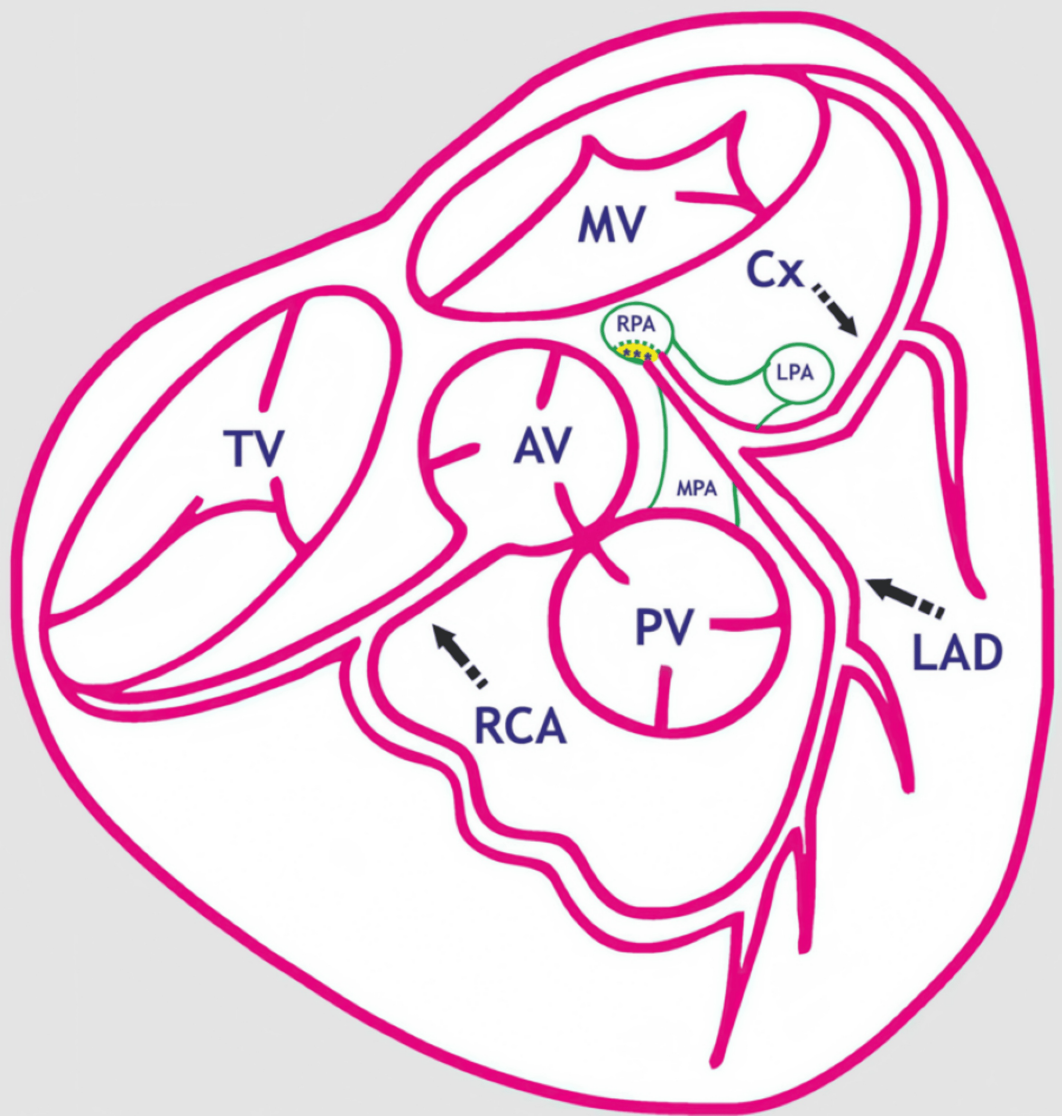
Diagrammatic representation of the abnormal origin of the LCA from the RPA (ALCARPA) in a transverse section of the heart. MV, mitral valve; AV, aortic valve; LAD, left anterior descending coronary artery; PV, pulmonic valve; Cx, circumflex coronary artery; RCA, right coronary artery; RPA, right pulmonary artery; TV, tricuspid valve; LPA, left pulmonary artery; MPA, main pulmonary artery; ***, intra-mural course Image Credits: Dr. Vishal V. Bhende

Less than 50 cases of ALCARPA have been documented [1,6,7]. ALCARPA may present concurrently with various congenital abnormalities of the heart, such as tetralogy of Fallot, aortic coarctation, ventricular septal defect, hypoplastic left heart syndrome, and double outlet right ventricle (Figure 3) [8].

Diagnosis can be established through ECHO if it reveals a significantly dilated RCA and an enlarged LV exhibiting global hypokinesia. ECHO may reveal strong, fast flow in the interventricular septum as a result of collateral circulation and reversed blood flow from the abnormal vessel back into the MPA. When diagnostic uncertainty persists, coronary angiography is recommended. Cardiac catheterization and ECHO help confirm the diagnosis and exclude other CA abnormalities, such as myocarditis or dilated cardiomyopathy [9]. Myocardial perfusion studies are selectively indicated for patients with significant global hypokinesia, particularly when heart transplantation is being considered.

If left untreated, ALCARPA may result in malignant ventricular arrhythmias that can be fatal. However, these arrhythmias typically resolve following the reestablishment of a dual-coronary system [10]. These findings highlight the critical nature of ischemia, regardless of whether symptoms are present, and support the need for prompt surgical intervention upon diagnosis, regardless of age or the extent of intracoronary collateralization [11].

Anomalous CA have been treated surgically using a variety of methods, such as baffle repair, direct aortic implantation, and ligation. Baffle repair is preferred when the anomalous vessel navigates a long intramural course within the PA wall. CA bypass grafting or subclavian-to-anomalous coronary artery anastomosis, which have been used for patients with ALCAPA, may be a last-resort option but are not desirable [12-15]. Unlike ALCAPA, Takeuchi tunneling is usually not feasible for patients with ALCARPA [16].

In most patients with ALCARPA, including those with an intramural segment within the aortic wall, the anomalous CA can safely be implanted into the aorta [6]. In rare instances, when this anomalous vessel traverses the aortic wall, alternative surgical approaches are needed to prevent CA damage.

Postoperatively, mechanical circulatory support may be required for children who cannot be weaned from CPB, especially patients in cardiogenic shock. Extracorporeal membrane oxygenation (ECMO) or the use of left ventricular assist devices could support LV recovery with favorable outcomes [17]. The various surgical strategies to tackle anomalous arising CA are represented in Table 1 [16,18-22].

**Table 1:** Various surgical strategies to tackle anomalous arising CA. LCA, left coronary artery; MPA, main pulmonary artery; CA, coronary arteries

Sr. No.	Author & Year	Number of Cases	Age in Years/Sex	Primary Presentation/Diagnosis	Surgical Technique Employed
01	Tashiro et al., 1993 [18]	2 adult patients	35/M; 68/F	ALCAPA	Left main coronary angioplasty using a transected MPA, side-to-side anastomosis of the aorta and newly created LCA, and direct anastomosis of the transected pulmonary artery without any prosthetic material
02	Ueyama et al., 1997 [19]	40/10,216 adult cardiac catheterization procedures	Adult patients	Anomalous circumflex CA (ACCAs)	Bypass grafting
03	Shetty et al., 2015 [20]	01	3 months/male, 3.5 kg	Anomalous single CA from MPA (ASCAPA)	A large surrounding button of the natural pulmonary artery wall was removed along with the aberrant CA's origin. Sutures made of continuous 7/0 polypropylene were used to mobilize, move, and implant it into the aortic anterior wall. The root defect of the MPA was repaired with an autologous pericardium patch
04	Bhende et al., 2021 [16]	01	5 months/male	ALCAPA; the LCA arises from the MPA's non-facing sinus	Takeuchi technique
05	Kaushik et al., 2022 [21]	01	1 year	Anomalous RCA originates from the RPA (ARCAPA)	Surgical intra-pulmonary baffle, the right coronary ostium is re-routed to the aortic root
06	George et al., 2024 [22]	01	5 months/male	ALCAPA – Left anterior non-facing sinus of MPA	LCA was reimplanted to the aorta using an extrapulmonary baffle made of an autologous pulmonary flap

## Conclusions

It is extremely uncommon and challenging for a congenital cardiac surgeon to diagnose and repair ALCARPA in conjunction with other cardiac abnormalities. Since echocardiographic results can occasionally be non-specific and even catheter diagnosis might be challenging, epicardial echocardiography is a crucial evaluation modality in the operating room.

Diagnosis of ALCARPA requires acute clinical awareness. The best repair method of ALCARPA is debatable in the literature; some studies support direct reimplantation of the coronary artery, while others support unroofing of the periaortic segment. Surgically establishing the transfer of the aberrant LCA to the aortic root using the dual-coronary system is the preferred method of correcting ALCARPA. An intramural course should be suspected for any abnormal LCA that is discovered in the vicinity of the right aspect of the MPA. To avoid unintentionally harming the intramural course, dissection between the aorta and MPA should be avoided or done very carefully.
